# Acceptability, fidelity and trial experience of four intervention components to support medication adherence in women with breast cancer: A process evaluation protocol for a pilot fractional factorial trial

**DOI:** 10.3310/nihropenres.13337.2

**Published:** 2023-04-24

**Authors:** Sophie M.C. Green, Louise H. Hall, Nikki Rousseau, David P. French, Christopher D. Graham, Michelle Collinson, Ellen Mason, Hollie Wilkes, Daniel Howdon, Robbie Foy, Rebecca Walwyn, Jane Clark, Catherine Parbutt, Erin Raine, Rachel Ellison, Jacqueline Buxton, Sally J. L. Moore, Galina Velikova, Amanda Farrin, Samuel G. Smith

**Affiliations:** 1 Leeds Institute of Health Sciences, University of Leeds, Leeds, LS29NL, UK; 2 Leeds Institute of Clinical Trials Research, University of Leeds, Leeds, LS29NL, UK; 3 Manchester Centre for Health Psychology, University of Manchester, Manchester, M13 9PL, UK; 4 Department of Psychology, Queen's University Belfast, Belfast, BT9 5BN, Ireland; 5 Department of Clinical and Health Psychology, Leeds Teaching Hospitals NHS Trust, Leeds, LS9 7TF, UK; 6 Medicines Management and Pharmacy Services, Leeds Teaching Hospitals NHS Trust, Leeds, LS9 7TF, UK; 7 Independent Researcher, Leeds, UK; 8 Leeds Institute of Medical Research at St James’s, University of Leeds, Leeds, LS9 7TF, UK; 9 Leeds Cancer Centre, Leeds Teaching Hospitals NHS Trust, St James’s University Hospital, Leeds, LS9 7TF, UK

**Keywords:** process evaluation, intervention fidelity, acceptability, trial experience, fractional factorial

## Abstract

**Background:**

The Refining and Optimising a behavioural intervention to Support Endocrine Therapy Adherence (ROSETA) programme has developed four intervention components aiming to improve medication adherence in women with early-stage breast cancer. These are (a) text messages, (b) information leaflet, (c) Acceptance and Commitment Therapy-based guided self-help (ACT), (d) side-effect management website. Guided by the Multiphase Optimisation Strategy, our pilot trial will use a fractional factorial design to evaluate the feasibility of undertaking a larger optimisation trial. The pilot will include a process evaluation to maximise learning regarding the fidelity and acceptability of the intervention components before proceeding with a larger trial. The trial process evaluation has three aims: to assess the (1) fidelity and (2) acceptability of the intervention components; and (3) to understand participant’s trial experience, and barriers and facilitators to recruitment and retention.

**Methods:**

The process evaluation will use multiple methods. Fidelity of the intervention components will be assessed using self-reported questionnaire data, trial data on intervention component adherence, and observations of the ACT sessions. Acceptability of the intervention components and trial experience will be explored using an acceptability questionnaire and interviews with patients and trial therapists. Trial experience will be assessed using a questionnaire and interviews with participants, while barriers and facilitators to recruitment and retention will be assessed using a questionnaire completed by research nurses and participant interviews. The pilot trial opened for recruitment on 20th May 2022 and was open at the time of submission.

**Conclusions:**

This process evaluation will provide information regarding whether the intervention components can be delivered with fidelity within a national healthcare setting and are acceptable to participants. We will also better understand participant experience in a pilot trial with a fractional factorial design, and any barriers and facilitators to recruitment and retention.

**Registration:**

ISRCTN registry ( ISRCTN10487576, 16/12/2021).

## Plain English summary

Background: The majority of women with early-stage breast cancer are recommended adjuvant endocrine therapy (AET) to reduce the chances of their cancer coming back. Many women given this medication don’t take it every day or stop taking it earlier than they should. We have developed four different interventions to help women take AET. These are; text messages reminding women to take AET; an information leaflet explaining how AET works and its benefits and side-effects; a therapy programme to reduce distress, consisting of five support sessions and four module booklets; and a website with strategies to manage AET side-effects. We are now testing whether these interventions can be delivered within the NHS in different combinations, in a small trial.

Study methods:

We have three aims:

1. To find out if the interventions can be given and are received in the way they were supposed to (fidelity).
2. To find out if the support received as part of the trial was acceptable to women with breast cancer (acceptability).
3. To find out what women’s experience was of taking part in the trial overall (trial experience).

To do this we will:

1. Interview participants to ask them how acceptable they found the interventions, what they understood, whether they used the interventions, and how they found participating in the trial.
2. Interview therapists who delivered the therapy programme to see if they delivered it as they were supposed to, and how they found delivering the intervention.
3. Ask participants to complete questionnaires about how acceptable the interventions were, and whether they read and used them.
4. Ask the staff involved in finding participants for the trial about challenges and improvements.

We will use what we find to make improvements in a future trial where we will test whether the interventions help women to take AET.

## Introduction

Breast cancer is the most common cancer in the UK ^1^. Adjuvant endocrine therapy (AET) is prescribed to women with oestrogen receptor-positive (ER+) breast cancer for 5–10 years to reduce recurrence and mortality ^2, 3^. However, up to three quarters of women do not adhere to AET, either taking medication inconsistently or stopping prematurely ^4– 6^. Suboptimal adherence can lead to increased risk of recurrence and mortality, reduced health related quality of life, and reduced quality adjusted life years ^7– 9^. Multiple factors affect non-adherence behaviours, and these are often described as intentional ( *e.g.*, not believing the medication is necessary, and side-effects), and unintentional ( *e.g.*, forgetting) ^10– 13^.

There is a lack of evidence for effective interventions to support medication adherence to AET, with most interventions focusing solely on written educational components, and not targeting the range of barriers to adherence ^14– 16^. Moreover, these interventions are typically evaluated using parallel group randomised controlled trials (RCTs). RCTs are able to evaluate whether an intervention package is more effective than a comparator, but they are unable to estimate the contributions of individual components, or the interactions between components. As such, intervention packages demonstrating a statistically significant effect in an RCT could contain ineffective or redundant components, reducing the efficiency of the overall intervention package ^17, 18^.

### The ROSETA programme

The Refining and Optimising a behavioural intervention to Support Endocrine Therapy Adherence (ROSETA) programme aims to develop and optimise an intervention package to support adherence to AET in women with early-stage breast cancer. The ROSETA programme is guided by the Multiphase Optimisation Strategy (MOST), which is a framework used to optimise multicomponent behavioural interventions ^17, 19, 20^. MOST consists of three stages; (1) preparation, in which intervention components and a conceptual model detailing proposed mechanisms of action are developed, and any pilot testing is carried out, (2) optimisation, in which highly efficient experimental designs, such as factorial designs, are used to estimate the main effects and interaction effects of individual intervention components to build an optimal intervention package, and (3) evaluation, in which the optimised intervention package is compared with a comparator, typically using a parallel groups RCT ^17, 19, 20^. 

**Figure 1.**
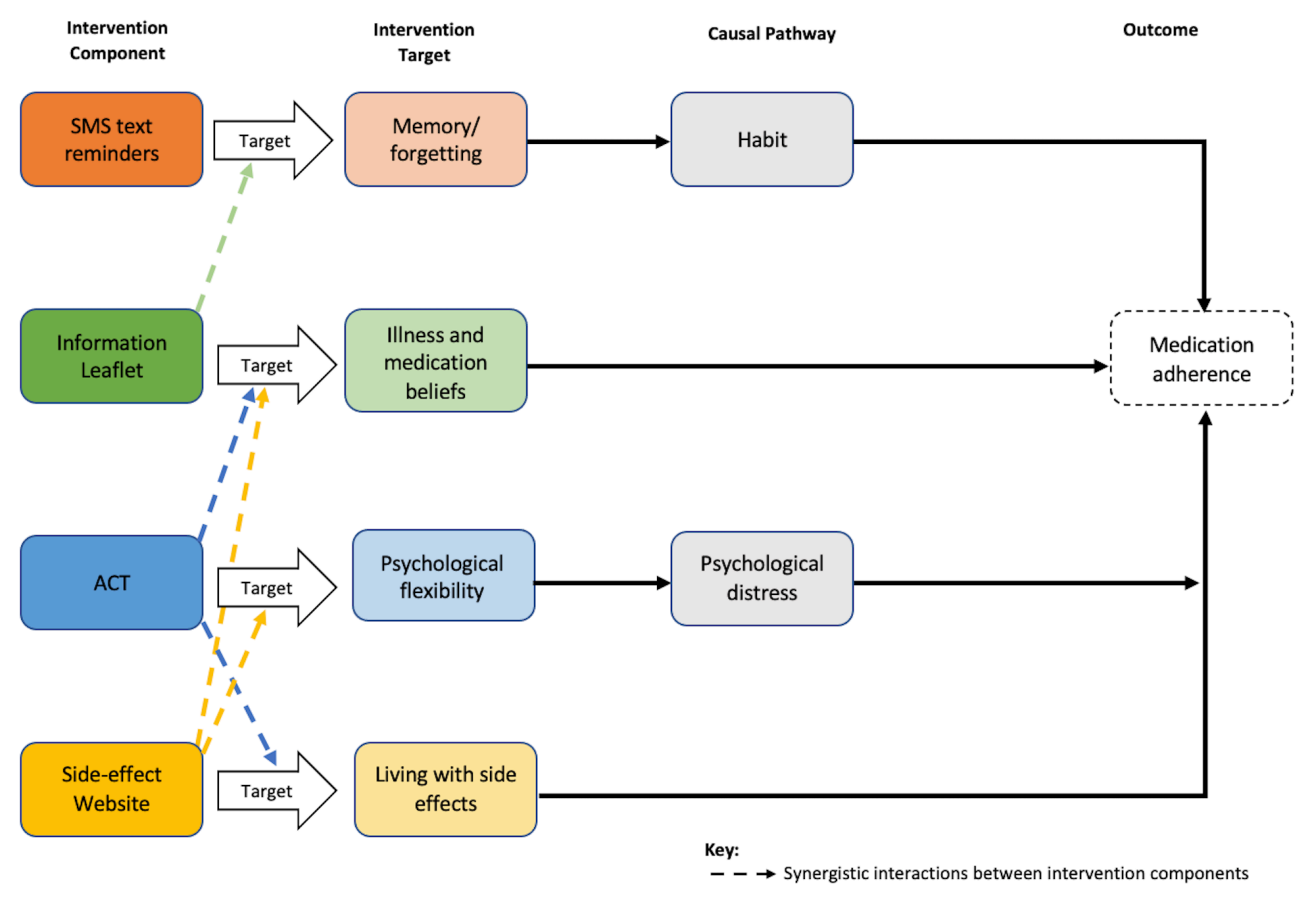
Conceptual model for the ROSETA intervention. This figure has been reproduced with permission from Green *et al.*, (2023) ^21^.

During the preparation phase of MOST, we combined the intervention mapping framework with MOST to develop four theory-based intervention components targeting distinct, unintentional and intentional barriers to AET adherence: (a) SMS messages to target forgetfulness; (b) information leaflet to target medication beliefs; (c) Acceptance and Commitment Therapy (ACT) guided self-help programme to increase psychological flexibility and reduce psychological distress; (d) side-effect self-management website to target AET side effects ^21^ ( Table 1). As part of this preparatory work, we developed a conceptual model for the intervention, detailing the mechanisms of action ( Figure 1). A full description of our approach to intervention development, along with detailed descriptions of the intervention components is available elsewhere ^21^.

**Table 1.** Summary of intervention components in the ROSETA pilot trial.

Component	Target	Description	Theoretical basis
SMS	Forgetfulness/habit formation	SMS messages will be sent over 4 months providing practical strategies to support regular medication taking each day. The messages will be sent daily for 2 weeks, twice weekly for 8 weeks and weekly for 6 weeks.	Habit theory
Information Leaflet	Medication beliefs	A written information leaflet containing five elements; an explanation of how AET works with diagrams to supplement, visual displays of the benefits of AET, accurate information about the side effects of AET, answers to common concerns about AET and quotes and pictures of breast cancer survivors.	Necessity Concerns Framework, Common Sense Model of Illness Representations
ACT	Psychological flexibility/psychological distress	A guided self-help intervention based on ACT principles involving four skills; mindfulness, unhooking, following values and living beyond labels. The modules consist of a participant booklet, home practice tasks and audio files. The modules are supported by five individual sessions with a psychologist; 1 x 15 minute opening session, 3 x 25 minute sessions following modules 1, 2 and 3, and 1 x 15 minute closing session following module 4.	ACT (based on relational frame theory)
Website	Side-effect self- management	A website containing strategies to self-manage common AET side effects including; arthralgia, fatigue, vulvovaginal symptoms, gastrointestinal symptoms, hot flushes, sleep difficulties. The website uses a rating system to summarise the strength of evidence for each strategy.	Informed by evidence of side-effect self- management strategies

Key: ROSETA= Refining and Optimising a behavioural intervention to Support Endocrine Therapy Adherence. SMS= Short Message Service. AET= adjuvant endocrine therapy. ACT= Acceptance and commitment therapy.

Experimental versions of the intervention components are currently being assessed for feasibility in an external, multi-site exploratory pilot trial, for which a detailed protocol has been published ^22^. This pilot trial is using a highly efficient 2 ^4-1^ fractional factorial design. Participants are randomised to one of eight experimental conditions, in which they may receive usual care plus a combination of intervention components. Each intervention component has two levels; on or off. Across five UK sites, the pilot trial will recruit approximately 80 women over 18 with stage I-IIIa breast cancer who have been prescribed AET and have completed curative hospital-based treatment. Follow up will be at 2 and 4 months post randomisation. The primary objectives of the pilot trial are to establish eligibility, recruitment, retention and follow up rates, intervention component adherence and availability and feasibility of outcome and process data (ISRCTN: 10487576).

### ROSETA pilot trial process evaluation

Process evaluations of complex interventions can maximise learning from trials, through investigating how an intervention was implemented, clarifying causal mechanisms and exploring contextual factors ^23^. As highlighted in the UK Medical Research Council’s (MRC) guidance, process evaluations can have differing functions dependent on the trial stage ^23^. In the context of a pilot trial, understanding the feasibility, fidelity and acceptability of an intervention is often the focus, falling within the implementation and context themes in MRC guidance ^23^.

The fractional factorial design in the ROSETA pilot trial is uncommon in healthcare research ^24^. When used with effect coding (-1, +1), factorial designs can efficiently estimate the main effects and interaction effects of multiple intervention components ^25^. However, this design requires multiple experimental conditions, thereby reducing the number of participants randomised to each experimental condition (the condition corresponds to a unique combination of the levels of intervention components). While adequate recruitment and retention of participants is challenging in all healthcare trials ^26, 27^, it is particularly important when using factorial designs, as empty cells may threaten the integrity of the trial ^17^, or increase complexity in analysis and interpretation. This trial design brings further uncertainties, such as whether multiple intervention components can be delivered with fidelity, participants are not unduly burdened with multiple intervention components, and whether the trial design is acceptable to, and understood by participants. Understanding participant trial experience and exploring barriers and facilitators to recruiting and retaining participants within a factorial design will provide important insights prior to proceeding with a larger optimisation trial. The Theoretical Domains Framework is a helpful tool to understand barriers and facilitators to recruitment and retention. The TDF synthesises 33 theories of behaviour change into factors that could influence behaviour, for example knowledge or available resources ^28^.

Intervention fidelity refers to whether an intervention component was implemented as originally intended ^29^. Fidelity assessments commonly focus solely on fidelity of delivery of an intervention, with less regard for the fidelity of training, receipt or engagement ^30, 31^. The US National Institute of Health Behaviour Change Consortium (NIHBCC) framework suggests fidelity as a multi-faceted construct and recommends five domains of fidelity that should be assessed; design, training, delivery, receipt and enactment ^32^. Assessing fidelity at multiple levels can guide targeting of efforts to improve fidelity and hence improve the internal and external validity of a larger trial ^31, 33, 34^.

Assessment of acceptability is also recommended in the pilot phase ^23, 35^. However, acceptability is frequently assessed as one singular construct, and the definition varies considerably ^36^. The Theoretical Framework of Acceptability (TFA) seeks to address this limitation by conceptualising acceptability as a multi-faceted construct consisting of seven components; affective attitude, burden, ethicality, intervention coherence, opportunity costs, perceived effectiveness and self-efficacy ^36^. While preliminary work has confirmed the prospective acceptability of the ROSETA intervention components, the pilot trial offers the chance to evaluate acceptability of the intervention components as experienced by intervention recipients, with the potential to improve acceptability prior to a larger optimisation trial.

## Protocol

### Aims and objectives

This process evaluation has three aims: to assess (1) the fidelity of the intervention components; (2) the acceptability of the intervention components; (3) participant’s experience, and barriers and facilitators to recruitment and retention in a fractional factorial trial. These aims address the implementation and contextual factors themes outlined in the MRC framework ^23^. The specific objectives for each aim are:

1. Fidelity; Establish the fidelity of each intervention component across five domains of fidelity, guided by the NIHBCC fidelity framework ^32^:
  a. Design. To establish to what extent the intervention components target the behaviour change techniques (BCTs) they are intended to, and to what extent the components are distinct from one another;
  b. Training. To evaluate the adequacy of therapist training for the delivery of the intervention;
  c. Delivery. To assess if each intervention was delivered as planned;
  d. Receipt. To evaluate if participants receive and understand the intervention components they were allocated to;
  e. Enactment. To understand the degree to which participants implement the intervention components in their daily life;

Identify barriers to the fidelity of training, delivery, receipt and enactment of the intervention components, including any influential contextual factors.

2. Acceptability:
  a. Assess the acceptability of each intervention component
3. Trial experience, recruitment and retention:
  a. Establish the barriers and facilitators to recruitment and retention of participants;
  b. Evaluate participant experience of trial participation.

### Design

We will use quantitative and qualitative methods to address the three main aims, nested within the ROSETA pilot trial. The process evaluation will involve assessments with three participant groups; participants of the ROSETA pilot trial, therapists delivering the ACT intervention component, and research nurses (RNs) involved in recruitment.

***Fidelity***. Fidelity will be assessed in relation to the intended design and delivery, across five domains using a mix of methods. Table 2 describes the data collection methods for each objective and intervention component, along with the time point at which the data will be collected.

**Table 2.** Summary of fidelity assessments.

Objective	Intervention Component	Data	Data source	Method of completion	Time point
**Design.** To establish to what extent the intervention components target the behaviour change techniques they are intended to, and to what extent the components are distinct from one another.	All	BCTs coded as present in each intervention component, based on the BCTTv1	Independent coder	Independent Coder	Once the trial has commenced
**Training.** To evaluate the adequacy of therapist training for the delivery of the intervention component.	ACT	ACT-FM competency assessment	Questionnaire	ACT trainer	Following therapists first ACT session
	ACT	Number of booster training sessions required	Questionnaire	ACT Trainer	Throughout trial
	ACT	Therapist’s view on ACT training	Semi-structured interview	Therapist	End of trial
**Delivery.** To assess if each intervention component was delivered as planner.	SMS	Delivery of SMS messages	Online System	-	Throughout trial
	SMS	Number of participants opting out	Online System	-	Throughout trial
	Information Leaflet	Number of information leaflets sent	Site Recorded	Research Nurse	Throughout trial
	ACT	Procedural fidelity checklist	Questionnaire	Therapist	After every ACT session
	ACT	ACT-FM therapist stance score	Questionnaire	External rater	End of trial
	ACT	Qualitative data	Semi-structured interview	Therapist	End of trial
	Website	Number of log in details sent	Site Recorded	Research Nurse	Throughout trial
**Receipt.** To evaluate if participants receive and understand the intervention components they were allocated to.	All	Self-reported receipt of intervention component	Questionnaire	Participant	4 months
	All	Self-reported reading of intervention component	Questionnaire	Participant	4 months
	ACT	Session attendance, number of cancelled or missed	CRF	Therapist	After every ACT session
	ACT	Engagement with ACT module materials	CRF	Therapist	After every ACT session
	ACT	Engagement with ACT audio files and home practice tasks	Questionnaire	Participant	4 months
	ACT	Engagement with ACT module materials	Questionnaire	External rater (10% of ACT recordings)	End of trial
	ACT	Printing of ACT module booklet	Questionnaire	Participant	4 months
	Website	Website usage	Website Online System	-	2 and 4 months
	All	Understanding and receipt of the intervention component	Qualitative interview	Participant	End of trial
**Enactment.** To understand the degree to which participants implement the intervention components in their daily life.	All	Qualitative data	Qualitative interview	Participant	End of trial
Identify barriers to the fidelity of training, delivery, receipt and enactment of the intervention components, including any influential contextual factors.	All	Qualitative data	Semi-structured interview	Participants and therapists	End of trial

Key: BCT= Behaviour Change Technique. BCTTv1 = Behaviour Change Technique Taxonomy version 1. ACT = Acceptance and Commitment Therapy. ACT-FM: Acceptance and Commitment Therapy Fidelity Measure. SMS = Short Message Service. CRF = Case Report Form.

Fidelity of design. We will assess whether the intervention components show fidelity to the intended design, in terms of BCTs. BCTs are considered ‘active ingredients’ of behaviour change interventions (e.g. problem solving, action planning). They can be coded using the behaviour change taxonomy version 1 (BCTTv1), which contains 93 BCTs ^37, 38^. The research team (SG, SS, LH and CG) have coded which BCTs are present for each intervention component ^21^. Two independent coders, external to the research team, will also code the intervention components. Coders will be provided with all intervention component materials and a coding manual and will be asked to identify which BCTs from the taxonomy are present in each intervention component. This process will be conducted once the trial has begun recruitment. The coders will have experience in using BCTs and will have completed BCTTv1 training. They will only code whether a BCT is present or absent, not the frequency of occurrence. Coding from the independent coders will be compared to the original coding ^21^, and an agreement coefficient will be calculated ^39^. All discrepancies will be discussed between one member of the research team (SG) and the two independent coders, and a final code list will be produced.

Fidelity of training (competency of delivery). The only intervention component to be delivered by healthcare professionals that requires training is the ACT component. Therapists will receive two half days of bespoke training from a clinical psychologist with expertise in ACT (CDG). To establish the adequacy of the training for delivery of the ACT component, a clinical psychologist (CDG) will assess the therapists recording of their first ACT session using the ACT Fidelity Measure (ACT-FM), which assesses therapist fidelity to ACT principles ^40^. The number of booster training sessions the therapists require will be monitored. Semi-structured interviews with the therapists will explore barriers to adequate training and scope for any improvements.

Fidelity of delivery. SMS receipt data will indicate whether a message has been successfully sent by the online system. The number of participants who opt-out of the SMS messages will be recorded. The number of information leaflets and website login details successfully sent to participants will be recorded by the site. For the ACT component, therapist scores on a procedural fidelity checklist will indicate which parts of the component were delivered ( *e.g.*, reflection on the utility, relevance and barriers to home practice tasks and introducing the following module). An expert ACT practitioner independent to the trial team will review 10% of all ACT session recordings using the ACT-FM, which assesses therapist fidelity to ACT principles. Reviewed recordings will include those conducted early and late in the trial and from all therapists involved.

Fidelity of receipt. Participants will be asked a single self-reported item to assess which intervention components they received. Self-reported engagement with the intervention component(s) will be collected, along with reasons for non-engagement. For the website, tracking data will be collected, including number of logins, time spent on website, pages visited and videos watched. Each participant will only be asked about the components they were randomised to receive. Semi-structured interviews will investigate fidelity of receipt and elicit any barriers or enablers to the receipt of all intervention components.

ACT therapists will report the number of sessions attended, cancelled or missed by each participant, and the participant’s engagement with module materials (participant manual, associated audio files and home practice tasks). Participant’s engagement with the module materials will also be assessed by an external reviewer assessing 10% of the ACT session recordings.

Fidelity of enactment. Semi-structured interviews will be used to assess the extent to which the participant uses the intervention component(s) in their day-to-day life, and any barriers or facilitators to this.

***Intervention component acceptability***. The assessment of acceptability of the intervention components will be guided by the TFA ^36^ ( Table 3). As described in Table 3, acceptability will be assessed using an acceptability questionnaire specific to each intervention component at the 4 month follow up ^41^, and semi-structured interviews with trial participants and therapists at the end of the trial.

**Table 3.** Summary of acceptability assessments.

Objective	Data	Data source	Method of completion	Time point
Assess the acceptability of each intervention component	Acceptability Questionnaire	Questionnaire	Participant	4 months
	Qualitative data	Semi-structured interview	Participant	End of trial
	Qualitative data	Semi-structured interview	Therapist	End of trial

***Trial experience, recruitment and retention***. A mix of methods will involve trial participants and RNs ( Table 4). Trial experience will be assessed in participants using a questionnaire assessing experience before, during and after the trial, a single item assessing overall trial acceptability, and during semi-structured interview with questions guided by the TFA ^36^. The assessment of the barriers and facilitators to recruitment and retention to the factorial trial will be guided by the TDF ^28^. Assessment will include one questionnaire for RNs exploring barriers and facilitators to recruitment, and interviews with participants with questions focusing on any barriers to participation and retention in the trial.

**Table 4.** Summary of trial experience, recruitment and retention assessments.

Objective	Data	Data source	Method of completion	Time point
Evaluate participant experience of trial participation	SPFQ	Questionnaire	Participant	Baseline, 2 months, 4 months
	Acceptability Questionnaire (single item)	Questionnaire	Participant	4 months
	Qualitative data	Semi-structured interview	Participant	End of trial
Establish the barriers and facilitators to recruitment and retention of participants	Recruitment Questionnaire	Questionnaire	Research Nurse	End of trial
	Qualitative data	Semi-structured interview	Participant	End of trial
	Qualitative data	Semi-structured interview	Research Nurse	End of trial

Key: SPFQ= Study Participant Feedback Questionnaire.

### Sampling and recruitment

***Trial participants***. Women over 18 years old taking AET (tamoxifen, raloxifene, anastrozole, letrozole or exemestane) for early stage (I to IIIa) breast cancer, who have completed their last hospital treatment within the previous 12 months, are eligible to be part of the ROSETA pilot trial ( Table 5). All trial participants will be asked to complete the quantitative assessments of the process evaluation at baseline, 2 and 4-month follow-up alongside the pilot trial assessments.

**Table 5.** Eligibility criteria for participation in the ROSETA pilot trial.

Inclusion Criteria	Exclusion Criteria
1. An informed consent form (signed and dated) 2. Capacity to provide informed consent 3. Women with early stage (1 to 3a) breast cancer according to the Tumour, Node, Metastasis (TNM) / American Joint Committee on Cancer (AJCC) staging system. *Note. Women being treated for a second primary breast cancer or a breast cancer local* * recurrence are eligible for the study, providing the most recent cancer is being treated with* * adjuvant endocrine therapy, and they meet all eligibility criteria. Women with bilateral breast* * cancer are permitted, providing at least one breast is affected by hormone receptor-positive* * disease.* 4. Aged ≥18 years old at time of screening for ROSETA’s pilot study 5. Have sufficient proficiency in English to be able to adhere to all intervention components and data collection required 6. Treated with curative intent 7. Completed their hospital-based treatment ( *e.g.*, surgery, radiotherapy and/or chemotherapy) for the current breast cancer within the last 12 months. *Note. Women are still eligible for the study if they are being treated with monoclonal* * antibody-based therapy such as trastuzumab, Kadcyla, pertuzumab, and Phesgo.* 8. Currently prescribed oral adjuvant Hormone Therapy (tamoxifen, raloxifene, anastrozole, letrozole, exemestane) 9. The participant is willing to complete the study questionnaires * 10. The participant is willing to be audio recorded during the therapy sessions * 11. The participant is willing and able to attend all ACT sessions either *via* video conference or telephone * 12. The participant is willing and able to complete home practice tasks * 13. Access to a mobile phone to receive SMS messages * 14. Willing to receive frequent SMS messages * 15. Access to a computer or smart device that can access the internet *	1. Stopped taking adjuvant hormone therapy if it is clinically contraindicated according to clinical recommendation 2. Women with Metastatic breast cancer 3. Currently or recently (last 6 months) involved in a similar research study where medication adherence is a primary outcome * 4. Currently attending psychotherapy/psycho-oncology/psychology/ counselling services, for any clinical reason * 5. Need for treatment for a severe mental health disorder or crisis, which is likely to interfere with participation ( *e.g.*, active psychosis, bipolar disorder, significant issues with addiction or self-harm or expressing active suicidal ideation with active plans and intent *) *Note, if concerned about the possible presence of risk of suicidal ideation* * with active plans and intent, then this can be assessed with the following* * questions, with patients ineligible if they answer ‘yes’ to 5c.* *Recently (in the last month):* * a. Have you had any thoughts about ending your life?* * b. (if yes) Have you thought about how you might go about it?* * c. (if yes) Do you intend to carry out this plan?* 6. Patients with a scheduled date for breast reconstruction surgery that is within their intervention delivery and follow-up period. *Note: Women* * planning to have a breast reconstruction but who have not scheduled a date* * for surgery are permitted.* 7. Auditory problems that would prevent the patient from participating in a telephone or video call, or hearing audio clips *

Items marked with * are collected via self-report. Key:ROSETA= Refining and Optimising a behavioural intervention to Support Endocrine Therapy Adherence. SMS= Short Message Service. ACT= Acceptance and commitment therapy. This table has been reproduced with permission from Smith *et al.*, (2023) ^22^.

Participation in the end of trial interview is optional. When providing initial consent at baseline, participants will be asked if they are willing to be contacted for an interview at the end of the trial. If willing, participants will be contacted with further information approximately three-months post-randomisation. Consent may be written or *via* telephone. We aim to interview at least one participant from each experimental condition (corresponding to unique combinations of intervention components) in which they receive at least one component. Maximum variation purposive sampling will aim to achieve a mix of participants above and below 50 years old. Participants will be interviewed until we have gained sufficient information power for each study aim (fidelity, acceptability and trial experience) ^42^. Information power will be discussed in regular team meetings to inform further sampling. The specific aims of this study, high specificity of the sample and use of established theoretical frameworks to inform the interview indicates a smaller sample size may be appropriate. The interviewer (SG) has had experience interviewing women with breast cancer taking AET for similar purposes ^43^ and therefore a good quality of dialogue is expected ^42^. However, this will require ongoing evaluation as this is dependent on the interviewer and participants. The analytic strategy will explore themes across cases, rather than individual in depth analysis, which is likely to require an increase in sample size. Thus, a small to moderate sample size is expected to be required ^42^.

***Intervention deliverers (ACT therapists)***. Therapists delivering the ACT component will be Health and Care Professions Council (HCPC) or United Kingdom Council for Psychotherapy (UKCP) registered band 7a or above practitioner psychologists, or psychotherapists. Once the site has opened to recruitment, the therapist will be sent an invitation letter, information sheet and consent form. All consenting therapists delivering the ACT intervention component will be asked to complete the quantitative aspect ( *e.g.*, procedural fidelity checklists, fidelity assessments), and to participate in an end of trial interview.

One month before the end of the intervention delivery period, one therapist from each site will be randomly selected to be interviewed from those that have consented. Further therapists will be interviewed until the sample holds sufficient information power or there are no new consenting therapists left to interview. Information power will be discussed regularly in team meetings to inform further sampling ^42^. Based on the specific study aims, high specificity of the sample, use of theoretical frameworks, expected good quality dialogue and cross-case analysis, a small to moderate sample size is expected ^42^.

***Research nurses***. All RNs (or equivalent) involved in the recruitment of participants to the ROSETA pilot trial will be invited to participate in the process evaluation. Once the site has opened to recruitment, the RN will be sent an invitation letter, information sheet and consent form. If they consent, they will be asked to complete a questionnaire regarding the barriers and facilitators to recruitment at the end of the trial recruitment period.

### Assessment measures

***Participant***

Adherence to intervention components. To assess fidelity of receipt of the intervention components, participants will be asked two self-report items for each intervention component they were allocated to receive. The questions ask whether they received each intervention component, and how much of the intervention component they read. In relation to the ACT component, participants will be asked two additional items asking how much of the home practice tasks they completed, and how many of the audio files they listened to. Responses are on a 3-point scale; “none”, “at least some” and “all of”.

Acceptability Questionnaire (AQ) ^41^. The acceptability questionnaire, based on the TFA, will assess the acceptability of the individual intervention components, modified for each component ^36, 41^. Three items have been removed (ethicality, self-efficacy and opportunity costs) to reduce participant burden for those allocated to multiple intervention components. Five items remain assessing general acceptability, affective attitude, burden, perceived effectiveness and intervention coherence. Participants will answer on a 5-point Likert Scale.

For participants randomised to the ACT component, 15 extra items ask specifically about the acceptability of elements of ACT sessions and format. For participants randomised to the SMS component one additional item asks about the acceptability of the frequency of the SMS messages. One open question asks for any further comments about the acceptability of intervention components. The ‘general acceptability’ item from the AQ will assess the acceptability of participants’ experience in the trial as a whole. Participants will answer on a 5-point Likert Scale.

Study Participant Feedback Questionnaire (SPFQ) ^44^. The SPFQ will assess participants’ experience throughout the trial. This has been modified for the current trial. At baseline, participants will be asked two items about information received prior to the trial. Participants will answer on a 5-point Likert Scale. At the 2-month follow up, participants will be asked to complete three yes/no items regarding their experience during the trial. At the 4-month follow up participants will complete two items about their overall satisfaction with their experience in the trial, answering on a 5-point Likert Scale.

***Therapist***

ACT Fidelity Measure (ACT-FM) ^40^. ACT-FM is a measure of therapist fidelity to ACT principles when delivering treatment ( *i.e.*, competency). The ACT-FM therapist stance subscale will be used to assess fidelity of training and delivery of the ACT component. Only the therapist stance subscale will be used, as this is the subscale most relevant to therapist delivery of the ACT component. Four items assess ACT-consistent behaviours, and three items assess ACT-inconsistent behaviours. Each item is scored on a 4-point scale (0–3). Scores can range from 0–9 on each subscale. A score of >4 on ACT consistent behaviours, and <5 on ACT inconsistent behaviours will be considered competent.

Procedural Fidelity Checklist. A procedural fidelity checklist for each individual session will assess fidelity of delivery of the ACT component. The checklist is designed specifically for the ACT component in ROSETA, for therapists to self-rate whether they undertook core intervention procedures, such as reflection on the skills exercises and home practice tasks. It includes eight items for session 1 and 2, seven items for session 3, six items for session 4, and four items for session 5. A percentage score is calculated for each session by dividing the score achieved by the maximum score achievable in that session and multiplying by 100. Additional items ask therapists to record to what extent a participant has engaged with the module materials (participant manual, home practice tasks and associated audio files). In each checklist, the therapists will also record the number of times the session has been cancelled.

***Research Nurse***

Barriers and facilitators to recruitment. RNs will complete a questionnaire to report their experiences of recruiting to a factorial trial. Questions are based on the TDF ^28^ and cover knowledge of the trial design, the influence of resources, social pressures and emotional factors on recruitment, their motivation levels in recruiting to the trial, their decision processes when contacting a patient to schedule a recruitment appointment, and any procedural changes that would facilitate recruitment.

### Qualitative interviews

A rapid qualitative approach will be used to ensure findings from the pilot trial process evaluation can inform the future optimisation trial in a timely manner ^45, 46^. Semi-structured interviews with trial participants and therapists will last up to one hour, will be conducted *via* telephone or videoconferencing software ( *e.g.*, Microsoft Teams), and will be recorded. During the interview, the interviewer will take notes. Immediately following each interview, the interviewer will complete an individual Rapid Research Evaluation and Appraisal Lab Rapid Assessment Procedure (RREAL RAP) sheet for the participant. RREAL RAP sheets are a two-column table summarising the information collected in an interview. Different RREAL RAP sheets will be used for therapists and participants.

***Trial participants***. Semi-structured interviews will assess fidelity of receipt and enactment, acceptability of the intervention components, and barriers and facilitators to recruitment and retention in the trial. Questions will be based on the NIH fidelity domains, the TFA, and the TDF. Interview guides have been reviewed and amended based on suggestions from our patient and public involvement group (participant interview questions can be found as *Extended data* ^47^).

***ACT therapists***. Semi-structured interviews with therapists will assess fidelity of training and delivery and any barriers to these, relationship building with participants in the intervention component, and acceptability of the ACT component (therapist interview questions can be found as *Extended data* ^47^). We will also explore any suggested improvements to the intervention component and establish whether it may be implementable in routine care in the NHS.

### Data analysis plan

***Quantitative analysis***

Intervention fidelity. Descriptive statistics will summarise the quantitative intervention component fidelity assessments ( Table 2). For the ACT component, these will also be presented by site. Specific to the fidelity of design assessment, the first-order agreement coefficient statistic (AC1) will estimate the interrater reliability between the independent coders and the BCTs that the components intended to target ^21, 39^. Strength of fidelity of design will be defined in terms of pre-established AC1 thresholds; <0.2=poor, 0.2 ≤ 0.4=fairly poor, >0.4 ≤0.6=moderate, >0.6 ≤0.8=good, >0.8 and ≤ 1=very good ^48^. Discrepancies will be resolved through discussions between SG and the coders.

Intervention component acceptability. Descriptive statistics will be calculated for the individual items on the acceptability questionnaire, and overall, for each intervention component. Additional questions regarding the acceptability of specific aspects of the ACT and SMS components will be summarised. All summaries will be presented by component. Missing data will be summarised.

Barriers and facilitators to recruitment and retention of participants. Descriptive statistics will be presented for the RN barriers to recruitment and retention questionnaire, alongside qualitative data from the questionnaire. Summaries will be presented overall and by site. Missing data will be summarised.

Participant experience of trial participation. Descriptive statistics will be presented (1) for overall trial experience, and split by experience before starting the trial, during the trial and at the end of the trial; (2) for the one item of the AQ aimed at assessing general acceptability of the trial overall, by experimental condition and by site. Missing data will be summarised.

***Qualitative analysis***. Individual RREAL RAP sheets completed immediately after the interview will be collated into higher level RREAL RAP sheets; one per component for data relating to intervention component acceptability and fidelity, one overall for data relating to participant trial experience, and one overall for therapist interview data ^45, 46, 49^. Regular team meetings will review emerging findings, changes to the interview schedule or RREAL RAP sheets, and data sampling. The interviews will be recorded and selectively transcribed verbatim. Specific quotes will be extracted from the transcripts and added to the RREAL RAP sheets to inform analysis. For each overall aim (fidelity, intervention component acceptability, and trial experience), the relevant quantitative and qualitative data will be combined narratively to determine whether the intervention components could be delivered with fidelity overall, whether each intervention component was acceptable to women with breast cancer, and whether the experience in the trial was acceptable to women with breast cancer, or if there were barriers to recruitment and retention within the pilot trial. Together these findings will be used to make improvements to the intervention components and trial processes, should these be necessary.

### Ethics and dissemination

The study has been approved by Wales Research Authority Research Ethics Committee 3 (21/WA/0322) and is a registered clinical trial (ISRCTN registry, ISRCTN10487576, 16/12/2021). It is sponsored by the University of Leeds ( governance-ethics@leeds.ac.uk). Amendments to the protocol will be submitted to the ethics committee, and if approved, communicated to research sites. Study findings will be disseminated through peer-reviewed publications. At the end of the trial, all data held by the CTRU and all trial data will then be securely archived at the University of Leeds for a minimum of 5 years. This paper is a summary of protocol version 3.0 (18/08/2022).

### Trial status

The study opened for recruitment on the 20th of May 2022 and was open at the time of submission.

## Discussion

This paper describes the design and methods of a process evaluation embedded in the ROSETA pilot trial. This process evaluation aims to investigate whether four intervention components can be delivered with fidelity, whether the intervention components are acceptable to participants and trial therapists, and to explore participant’s experiences in the trial along with barriers and facilitators to recruitment and retention. Given there is little guidance available for the conduct of process evaluations within factorial trial designs, our protocol may guide others seeking to conduct similar process evaluations.

The results of this process evaluation will be used to adapt and improve the intervention components where necessary, should progression criteria to a larger optimisation trial be met. Our progression criteria to progress to a full optimisation trial are based on predefined criteria for consent rates, intervention component adherence, and availability of outcome data. These are described in detail elsewhere ^22^. The full optimisation trial would aim to identify the most effective combination of intervention components to support medication adherence in women with early stage breast cancer, without exceeding a pre-specified cost of £3397 per patient, based on health economic modelling ^21^.

This process evaluation has the potential to highlight key issues in the specific intervention components in terms of fidelity and acceptability, and regarding the overall trial design and its implementation within an NHS setting. As factorial designs are relatively uncommon in behavioural trials, we hope that this process evaluation can offer useful insights for others.

## Data Availability

No data are associated with this article. Open Science Framework: ROSETA Pilot Trial Process Evaluation. https://doi.org/10.17605/OSF.IO/8DWRN ^47^. The project contains the following extended data: Participant Interview Guide Therapist Interview Guide Data are available under the terms of the Creative Commons Zero "No rights reserved" data waiver (CC0 1.0 Public domain dedication).
