# Supplementary material for: Acceptability, fidelity and trial experience of four intervention components to support medication adherence in women with breast cancer: A process evaluation protocol for a pilot fractional factorial trial
Source: NIHR Open Res. 2023 Apr 24;3:3. Originally published 2023 Jan 20. [Version 2] doi: 10.3310/nihropenres.13337.2 (PMC10593334; doi:10.3310/nihropenres.13337.2)
Supplement: Peer review history

# Reviewer report: Reviewer response for version 2

**DOI:** 10.3310/nihropenres.14552.r29195

**Published:** 2023-05-15

**Authors:** Molly Byrne

**Affiliations:** 1 Health Behaviour Change Research Group, School of Psychology, National University of Ireland, Galway, Galway, Ireland

Many thanks to the authors for their thorough considerations of my recommendations and for the amendments. I am now happy to approve the paper.

Is the study design appropriate for the research question?

Yes

Is the rationale for, and objectives of, the study clearly described?

Yes

Are sufficient details of the methods provided to allow replication by others?

Yes

Are the datasets clearly presented in a useable and accessible format?

Not applicable

Reviewer Expertise:

NA

I confirm that I have read this submission and believe that I have an appropriate level of expertise to confirm that it is of an acceptable scientific standard.

# Reviewer report: Reviewer response for version 2

**DOI:** 10.3310/nihropenres.14552.r29196

**Published:** 2023-05-09

**Authors:** Thomas Mills

**Affiliations:** 1 PHIRST South Bank, London South Bank University, London, England, UK

This is a much-improved manuscript: inclusion of the conceptual model helps convey what the intervention targets.

 I still don’t feel that the Plain English summary sets up the study as the authors are aiming to achieve more than “find out” stuff, and will be doing more than interviewing and asking. There’s a balance between making papers accessible and dumbing down and I don’t think the authors are striking it here. This is a matter of style however.

Is the study design appropriate for the research question?

Yes

Is the rationale for, and objectives of, the study clearly described?

Partly

Are sufficient details of the methods provided to allow replication by others?

Yes

Are the datasets clearly presented in a useable and accessible format?

Yes

Reviewer Expertise:

The qualitative component of process evaluations.

I confirm that I have read this submission and believe that I have an appropriate level of expertise to confirm that it is of an acceptable scientific standard.

# Reviewer report: Reviewer response for version 1

**DOI:** 10.3310/nihropenres.14463.r29031

**Published:** 2023-03-21

**Authors:** Thomas Mills

**Affiliations:** 1 PHIRST South Bank, London South Bank University, London, England, UK

Technically speaking, this is well thought out and looks to be an excellent process evaluation. One thing I’d like the authors to consider is *why* they are doing this. The initial set of aims needs developing: surely an aim here is to facilitate research-informed improvements to the intervention components ahead of the trial? I also feel there’s a gap pertaining to this aim in the protocol whereby the researchers draw on the findings to inform intervention changes, as well as their use of analytical frameworks to guide this: the TDF framework is mentioned on occasions but only in passing. A final section/sub-section after, or at the end of the data analysis section to outline a proposed plan for pulling it all together would add the cherry onto the cake, so to speak.

**Study methods**

- The aims need further development. I suggest having a set of aims and a set of objectives. Also, if the first set of aims is numbered, could the second set be letter-ed, so as to differentiate between them further?
- Aims 2 and 3 are similar – exploring women’s acceptability and experiential outcomes. I wonder if there is another aim, which is alluded to elsewhere in the paper, which is to use the process findings to refine intervention components ahead of the full trial.
- In the “To do this we will” section, 3. implies that intervention components are all readable, when therapy sessions are planned also. All of these points relate to data collection activities: I presume you’ll be analysing the data too, to fulfil the aims? Key analytical strategies could be included here.

**Roseta process evaluation intro**

- No mention of analytical strategy/data analysis – e.g., TDF framework

**Protocol – Aims and objectives**

- How do these, new aims relate to the aims stated in the study methods? These different sets of aims need to be linked.

**Design**

- Fidelity – another introduction to the five domains – it feels like you’re going backward because the domains have been introduced already: focus this sentence on describing Table 2 in more detail.

**Fidelity design**

- Example BCTs would help the reader - possibility of a logic model to show the causal assumptions?

**Fidelity of enactment**

- Ditto – text could focus describing Table 3 in more detail.

**Trial experience, retention and recruitment**

- First mention of TDF framework – this is important because it will help the researchers understand the data and highlight areas of improvement

**Qualitative interviews**

- Why will the interviewer be taking notes during the interview? This risks obstructing the flow of the interview. The interview will be recorded. I suggest the interviewer write up the interview immediately after the interview, using the recording as a guide; then check the summary against the transcript, once the recording is transcribed.

Is the study design appropriate for the research question?

Yes

Is the rationale for, and objectives of, the study clearly described?

Partly

Are sufficient details of the methods provided to allow replication by others?

Yes

Are the datasets clearly presented in a useable and accessible format?

Yes

Reviewer Expertise:

The qualitative component of process evaluations.

I confirm that I have read this submission and believe that I have an appropriate level of expertise to confirm that it is of an acceptable scientific standard, however I have significant reservations, as outlined above.

# Response

**Published:** 2023-04-13

**Authors:** Sophie Green

**Affiliations:** University of Leeds, UK

Technically speaking, this is well thought out and looks to be an excellent process evaluation. One thing I’d like the authors to consider is *why* they are doing this.

 Thank you for taking the time to review our manuscript and for your constructive comments.

**The initial set of aims needs developing: surely an aim here is to facilitate research-informed improvements to the intervention components ahead of the trial? I also feel there’s a gap pertaining to this aim in the protocol whereby the researchers draw on the findings to inform intervention changes, as well as their use of analytical frameworks to guide this: the TDF framework is mentioned on occasions but only in passing.**

 RESPONSE: In line with our decision to undertake an external pilot trial, as opposed to a feasibility trial, using the findings from the process evaluation to refine the intervention components ahead of the optimisation trial was not a main aim of the research. The aims were to determine the acceptability of the intervention components, determine if they could be delivered with fidelity and to assess women’s experience within the trial. As such, this process evaluation contributes towards the broader goal of determining if a larger trial to optimise these components is feasible to undertake. We will make some minor amendments to the intervention components if it will improve their acceptability, fidelity or women’s trial experience, but this was not the primary purpose of doing a pilot trial with an embedded process evaluation.

 In terms of our used of analytical frameworks, we have now added an introduction to the TDF in the introduction. In the introduction we had already introduced the other two theoretical frameworks we are using; the theoretical framework of acceptability and the five domains of fidelity. In the ‘design’ section in the methods, we then explain which framework we are using for each aim of the process evaluation. The five domains of fidelity is being used to address the fidelity aim (aim 1), the theoretical framework of acceptability is being used to determine acceptability of the intervention components (aim 2), and the TDF is being used to assess participant trial experience and barriers and facilitators to recruitment and retention (aim 3).

**A final section/sub-section after, or at the end of the data analysis section to outline a proposed plan for pulling it all together would add the cherry onto the cake, so to speak.**

 RESPONSE: We have added an additional paragraph into the data analysis section as suggested to outline our proposed plan for pulling the quantitative and qualitative data together for each aim.

 Methods: “For each overall aim (fidelity, intervention component acceptability, and trial experience), the relevant quantitative and qualitative data will be combined narratively to determine whether the intervention components could be delivered with fidelity overall, whether each intervention component was acceptable to women with breast cancer, and whether the experience in the trial was acceptable to women with breast cancer, or if there were barriers to recruitment and retention within the pilot trial. Together, these findings will be used to make improvements to the intervention components and trial processes, should these be necessary.”

**Study methods**

- **The aims need further development. I suggest having a set of aims and a set of objectives. Also, if the first set of aims is numbered, could the second set be letter-ed, so as to differentiate between them further?**

RESPONSE: This comment is in reference to the aims within the plain English summary/ abstract of the work, with a word limit of 300 words. We therefore request that we do not go into detail about the additional objectives within each aim (as we also have not done in the scientific abstract), as there is insufficient space to include these here. In the main body of the paper, we have included aims, and specific objectives for each aim, using numbering and lettering to differentiate between these.

- **Aims 2 and 3 are similar – exploring women’s acceptability and experiential outcomes. I wonder if there is another aim, which is alluded to elsewhere in the paper, which is to use the process findings to refine intervention components ahead of the full trial.**

RESPONSE: Aim 2 refers to the acceptability specifically of each of the four intervention components, while aim 3 refers to women’s experience within the trial in relation to the novel trial design we are using, and general trial processes, such as the length of questionnaires. We have made some amendments to the wording of these aims in the plain English summary to differentiate them further.

 Please see our earlier response with regard to including an additional aim.

  “Plain English Summary Aims:

 2. To find out if the support received as part of the trial was acceptable to women with breast cancer (acceptability).

 3. To find out what women’s experience was of taking part in the trial overall (trial experience).”

- **In the “To do this we will” section, 3. implies that intervention components are all readable, when therapy sessions are planned also. All of these points relate to data collection activities: I presume you’ll be analysing the data too, to fulfil the aims? Key analytical strategies could be included here.**

RESPONSE: As part of the therapy intervention component, there are module booklets, which are the readable element that would be assessed here; we have clarified this in the background section of the plain English summary where we describe the intervention components, and we have included this information in Table 1, where we provide a full description of the intervention components.

 We have not included the analysis strategies here as this is the plain English summary with a small word limit, and we have also not included analytic strategies in the scientific abstract. We are using a range of different analysis approaches for the different aims and quantitative and qualitative data in this process evaluation, and therefore we feel that summarising these analytical methods within this plain English summary would add confusion more than it would provide clarity. We have described our planned analysis in detail in the main paper, under the heading “data analysis plan”.

**Roseta process evaluation intro**

- **No mention of analytical strategy/data analysis – e.g., TDF framework**

RESPONSE: We have now added in a description of the TDF into the introduction in the relevant section. The other frameworks we are using (theoretical framework of acceptability, and fidelity domains framework) are already referred to in the introduction. We have not included specific details of data analysis as we feel this is more appropriate in the methods section.

 Introduction: “The Theoretical Domains Framework is a helpful tool to understand barriers and facilitators to recruitment and retention. The TDF synthesises 33 theories of behaviour change into factors that could influence behaviour, for example knowledge or available resources (36).”

**Protocol – Aims and objectives**

- **How do these, new aims relate to the aims stated in the study methods? These different sets of aims need to be linked.**

RESPONSE: These are not new aims; the aims in the ‘study methods’ the reviewer refers to, are the aims written in a plain English summary. In both sets of aims, (1) refers to investigating intervention fidelity, (2) refers to intervention acceptability, and (3) refers to trial experience, which are the 3 overall aims of this process evaluation. These have been listed in the same order each time to indicate that they are the same aims. In the plain English summary we have now added detail in brackets to make the link clearer to the aims in the main text. 

**Design**

- **Fidelity – another introduction to the five domains – it feels like you’re going backward because the domains have been introduced already: focus this sentence on describing Table 2 in more detail.**

RESPONSE: We have revised this section you mention, focusing on describing Table 2 rather than referring to the five domains.

 Methods: “Fidelity will be assessed in relation to the intended design and delivery, across five domains using a mix of methods. Table 2 describes the data collection methods for each objective and intervention component, along with the time point at which the data will be collected.”

**Fidelity design**

- **Example BCTs would help the reader - possibility of a logic model to show the causal assumptions?**

RESPONSE: We have now added in two example BCTs to the text, and have included our conceptual model as a figure within the paper (Figure 1), which details our proposed mechanisms of action for each intervention component. We have also referenced our intervention development paper in the introduction, which contains the full list of BCTs we have coded for each intervention component.

 Introduction: “A full description of our approach to intervention development, along with detailed descriptions of the intervention components is available elsewhere (14). As part of this preparatory work, we developed a conceptual model for the intervention, detailing the mechanisms of action (Figure 1).”

 Methods: “BCTs are considered ‘active ingredients’ of behaviour change interventions (e.g. problem solving, action planning).”

**Fidelity of enactment**

- **Ditto – text could focus describing Table 3 in more detail.**

RESPONSE: Here, we assume the reviewer is referring to the “Intervention component acceptability” section which relates to Table 3 mentioned, rather than the fidelity of enactment section referred to above. We have kept the sentence referring to the TFA here, as it is the first time we explicitly state that we are using the TFA (we only introduce the framework in the introduction). However, we have added an additional sentence describing Table 3 as suggested by the reviewer.

 Methods: “The assessment of acceptability of the intervention components will be guided by the TFA (30)(Table3). As described in Table 3, acceptability will be assessed using an acceptability questionnaire specific to each intervention component at the 4 month follow up (35), and semi-structured interviews with trial participants and therapists at the end of the trial.”

**Trial experience, retention and recruitment**

- **First mention of TDF framework – this is important because it will help the researchers understand the data and highlight areas of improvement**

RESPONSE: We have now introduced the TDF earlier in the manuscript, in the introduction, in line with the other theoretical frameworks we are using in this work (theoretical framework of acceptability, and five domains of fidelity).

 Introduction: “The Theoretical Domains Framework is a helpful tool to understand barriers and facilitators to recruitment and retention. The TDF synthesises 33 theories of behaviour change into factors that could influence behaviour, for example knowledge or available resources (36).”

**Qualitative interviews**

- **Why will the interviewer be taking notes during the interview? This risks obstructing the flow of the interview. The interview will be recorded. I suggest the interviewer write up the interview immediately after the interview, using the recording as a guide; then check the summary against the transcript, once the recording is transcribed.**

RESPONSE: The interviewer will be taking notes during the interviews as this is part of the recommended process in rapid approaches. We have previously used this method and did not find it disrupted the interviews. The note taking will not be extensive and we will make every effort to ensure it does not disrupt the flow of the interview. Using the recording as a guide to write up the interview would take a substantial period of time to re listen to the interview, and would not fit with the rapid analysis approach that we are taking.

# Reviewer report: Reviewer response for version 1

**DOI:** 10.3310/nihropenres.14463.r28987

**Published:** 2023-02-14

**Authors:** Molly Byrne

**Affiliations:** 1 Health Behaviour Change Research Group, School of Psychology, National University of Ireland, Galway, Galway, Ireland

This is an excellent paper which I enjoyed reading. The team are commended for this ambitious and important programme of research. They are addressing a clinically important question. The paper is well-written and describes a novel trial design which has the potential to address important questions about active ingredients of complex interventions.

 I have a few suggestions which I believe would improve the proposal:

1. Introduction: There is a lack of discussion and information about what we know already in relation to improving medication adherence. The research is presented in a bit of a vacuum, and would be strengthened by including a little more detail summarising what is already known, on which this research is building.
2. Introduction: The team are employing a novel trial design, using MOST, or a pilot trial with a fractional factorial design. This may be new to many readers. It would be interesting and helpful to readers if the authors could include some comment of where a MOST trial sits in the intervention development and evaluation process. What would the next steps of this study be? An full scale optimisation trial? What would the aims of this trial be? Where is this work going? Some discussion of these important questions would really strengthen this paper and help move along discussions about the place of novel trial designs like these in the development/evaluation/implementation pipeline.
3. One of the key steps in an optimisation trial is developing a conceptual model of the intervention, detailing the components and proposed mechanisms of action. Did the team develop a conceptual model for this intervention? It would be helpful to readers to see that.
4. Related to point 3., there is very little in the paper about the intervention components and their behaviour change techniques. Can the authors include any more detail about the intervention components? Is there any theoretical rationale for the intervention? This discussion is very limited in the introduction.
5. The proposed timeframe for follow up (4 months) seems short. Is there a clinical rationale for selecting this timeframe? Would this be the proposed timeframe in a future optimisation trial?
6. There is not mention of economic considerations or an economic evaluation of the interventions. Is this being included in the work? The intervention components appear very variable in terms of cost, which is an important consideration in the evaluation of the interventions. Are there plans to consider this aspect in furfure research?
7. Discussion: Can the authors clarify the ‘progression criteria’, which referred to in the Discussion section.

Is the study design appropriate for the research question?

Yes

Is the rationale for, and objectives of, the study clearly described?

Yes

Are sufficient details of the methods provided to allow replication by others?

Yes

Are the datasets clearly presented in a useable and accessible format?

Not applicable

Reviewer Expertise:

Behaviour science; Health behaviour change research

I confirm that I have read this submission and believe that I have an appropriate level of expertise to confirm that it is of an acceptable scientific standard, however I have significant reservations, as outlined above.

# Response

**Published:** 2023-04-13

**Authors:** Sophie Green

**Affiliations:** University of Leeds, UK

**This is an excellent paper which I enjoyed reading. The team are commended for this ambitious and important programme of research. They are addressing a clinically important question. The paper is well-written and describes a novel trial design which has the potential to address important questions about active ingredients of complex interventions. I have a few suggestions which I believe would improve the proposal:**

 Thank you very much for taking the time to review our work and for your helpful comments to improve the manuscript.

**Introduction: There is a lack of discussion and information about what we know already in relation to improving medication adherence. The research is presented in a bit of a vacuum, and would be strengthened by including a little more detail summarising what is already known, on which this research is building.**

 RESPONSE: We have added an additional paragraph in the introduction (2 ^nd^ paragraph) to explain that interventions within this field are largely ineffective. We have additionally explained the limitations of randomised controlled trials that are typically used to evaluate these interventions, as we are building upon this through using the multiphase optimisation strategy within our research.  

 Introduction: “There is a lack of evidence for effective interventions to support medication adherence to AET, with most interventions focusing solely on written educational components, and not targeting the range of barriers to adherence (46-48). Moreover, these interventions are typically evaluated using parallel groups randomised controlled trials (RCTs). RCTs are able to evaluate whether an intervention package is more effective than a comparator, but they are unable to estimate the contributions of individual components, or the interactions between components. As such, intervention packages demonstrating a statistically significant effect in an RCT could contain ineffective or redundant components, reducing the efficiency of the overall intervention package (16, 49).

**Introduction: The team are employing a novel trial design, using MOST, or a pilot trial with a fractional factorial design. This may be new to many readers. It would be interesting and helpful to readers if the authors could include some comment of where a MOST trial sits in the intervention development and evaluation process. What would the next steps of this study be? An full scale optimisation trial? What would the aims of this trial be? Where is this work going? Some discussion of these important questions would really strengthen this paper and help move along discussions about the place of novel trial designs like these in the development/evaluation/implementation pipeline.**

 RESPONSE: We have added in additional explanation of the MOST framework, and the three main stages, to demonstrate where optimisation sits within the development and evaluation process. We have also made it clearer that this work falls within the preparation phase of MOST. We have now added additional detail regarding the next stages of this work in the discussion section, as that is where we felt it was most relevant. This includes an explanation of our progression criteria to move to the larger optimisation trial, and what the aim of the optimisation trial would be.****

 Introduction: “The Refining and Optimising a behavioural intervention to Support Endocrine Therapy Adherence (ROSETA) programme aims to develop and optimise an intervention package to support adherence to AET in women with early-stage breast cancer. The ROSETA programme is guided by the Multiphase Optimisation Strategy (MOST), which is a framework used to optimise multicomponent behavioural interventions (15-17). MOST consists of three stages; (1) preparation, in which intervention components and a conceptual model detailing proposed mechanisms of action are developed, and any pilot testing is carried out, (2) optimisation, in which highly efficient experimental designs, such as factorial designs, are used to estimate the main effects and interaction effects of individual intervention components to build an optimal intervention package, and (3) evaluation, in which the optimised intervention package is compared with a comparator, typically using a parallel groups RCT (15-17).

 During the preparation phase of MOST, we combined the intervention mapping framework with MOST to develop four theory-based intervention components targeting distinct, unintentional and intentional barriers to AET adherence: (a) SMS messages to target forgetfulness; (b) information leaflet to target medication beliefs; (c) Acceptance and Commitment Therapy (ACT) guided self-help programme to increase psychological flexibility and reduce psychological distress; (d) side-effect self-management website to target AET side effects (14)(Table1). As part of this preparatory work, we developed a conceptual model for the intervention, detailing the mechanisms of action (Figure 1). A full description of our approach to intervention development, along with detailed descriptions of the intervention components is available elsewhere (14).”

 Discussion: “The results of this process evaluation will be used to adapt and improve the intervention components where necessary, should progression criteria to a larger optimisation trial be met. Progression criteria to progress to a full optimisation trial are based on predefined criteria for consent rates, intervention component adherence, and availability of outcome data. These are described in detail elsewhere (45){Smith, 2023 #560}. The full optimisation trial would aim to identify the most effective combination of intervention components to support medication adherence in women with early stage breast cancer, without exceeding a pre-specified cost of £3397 per patient, based on health economic modelling (14).”

**One of the key steps in an optimisation trial is developing a conceptual model of the intervention, detailing the components and proposed mechanisms of action. Did the team develop a conceptual model for this intervention? It would be helpful to readers to see that.**

 RESPONSE: We did develop a conceptual model for the intervention, which we have published elsewhere and have referenced in the current paper (Green et al., 2022, BMC Health Services Research). We have now included our conceptual model as a figure in the present paper (Figure 1), and have referred readers to this in the introduction.

**Related to point 3., there is very little in the paper about the intervention components and their behaviour change techniques. Can the authors include any more detail about the intervention components? Is there any theoretical rationale for the intervention? This discussion is very limited in the introduction.**

 RESPONSE: We have now made it clear that the intervention components were developed based on psychological theory in the text, and we have included details of this in Table 1, alongside a description of the intervention components. We undertook an extensive intervention development process, which has been published elsewhere, and we have now made this more explicit to the reader here. Included in this previous publication is a full list of BCTs that we coded in each of our intervention components.

 Introduction: “During the preparation phase of MOST, we combined the intervention mapping framework with MOST to develop four theory-based intervention components targeting distinct, unintentional and intentional barriers to AET adherence: (a) SMS messages to target forgetfulness; (b) information leaflet to target medication beliefs; (c) Acceptance and Commitment Therapy (ACT) guided self-help programme to increase psychological flexibility and reduce psychological distress; (d) side-effect self-management website to target AET side effects (14)(Table1). As part of this preparatory work, we developed a conceptual model for the intervention, detailing the mechanisms of action (Figure 1). A full description of our approach to intervention development, along with detailed descriptions of the intervention components is available elsewhere (14).”

**The proposed timeframe for follow up (4 months) seems short. Is there a clinical rationale for selecting this timeframe? Would this be the proposed timeframe in a future optimisation trial?**

 RESPONSE: A short follow up was chosen in this pilot trial for a number of reasons. Firstly, retention was not included as part of the progression criteria to a full optimisation trial, and therefore we did not need to have a long follow up to assess this. The progression criteria (based on consent rates, intervention component adherence and availability of outcome data) are all based on short term outcomes, so a longer term follow up was not required. Finally, the funding structure for this work means that intervention development, a pilot trial and a full optimisation trial with a 12 month follow up are taking place within 6 years; therefore a shorter follow up period in the pilot trial felt appropriate to allow for this body of work to be completed within the timeframe.****

**There is not mention of economic considerations or an economic evaluation of the interventions. Is this being included in the work? The intervention components appear very variable in terms of cost, which is an important consideration in the evaluation of the interventions. Are there plans to consider this aspect in furfure research?**

 RESPONSE: We are planning an economic analysis in the larger optimisation trial, for which we will use self-reported data, alongside NHS reference costs and costs from the Personal Social Services Research Unit, who estimate unit costs for healthcare professionals and services in the NHS. The economic analysis will form part of the main trial paper, as it is not considered part of the process evaluation, and therefore we have not included it within this protocol. However, we have now included our optimisation objective for the full optimisation trial in the discussion, which highlights that we are considering cost in the optimisation of the intervention package.

 Discussion: “The full optimisation trial would aim to identify the most effective combination of intervention components to support medication adherence in women with early stage breast cancer, without exceeding a pre-specified cost of £3397 per patient, based on health economic modelling (14).”

**Discussion: Can the authors clarify the ‘progression criteria’, which referred to in the Discussion section.**

 RESPONSE: We have now added in a description of the progression criteria for this pilot trial, along with a reference to the trial protocol where progression criteria are described in greater detail.

 Discussion: “The results of this process evaluation will be used to adapt and improve the intervention components where necessary, should progression criteria to a larger optimisation trial be met. Progression criteria to progress to a full optimisation trial are based on predefined criteria for consent rates, intervention component adherence, and availability of outcome data. These are described in detail elsewhere (45){Smith, 2023 #560}.”
